# Gold Nanobiosensor Based on the Localized Surface Plasmon Resonance is Able to Diagnose Human Brucellosis, Introducing a Rapid and Affordable Method

**DOI:** 10.1186/s11671-021-03600-4

**Published:** 2021-09-16

**Authors:** Sina Vakili, Mohammad Samare-Najaf, Amirreza Dehghanian, Amir Tajbakhsh, Hassan Askari, Reza Tabrizi, Mahdiyar Iravani Saadi, Ahmad Movahedpour, Marzieh Alizadeh, Ali Samareh, Saeed Taghizadeh, Saam Noroozi

**Affiliations:** 1 Infertility Research Center, Shiraz University of Medical Sciences, Shiraz, Iran; 2 Biochemistry Department, School of Medicine, Shiraz University of Medical Sciences, Shiraz, Iran; 3 Trauma Research Center, Shiraz University of Medical Sciences, Shiraz, Iran; 4 Molecular Pathology and Cytogenetics Division, Department of Pathology, Shiraz University of Medical Sciences, Shiraz, Iran; 5 Pharmaceutical Sciences Research Center, Shiraz University of Medical Sciences, Shiraz, Iran; 6 Gastroenterohepatology Research Center, Shiraz University of Medical Sciences, Shiraz, Iran; 7 Noncommunicable Diseases Research Center, Fasa University of Medical Sciences, Fasa, Iran; 8 Hematology Research Center, Shiraz University of Medical Sciences, Shiraz, Iran; 9 Department of Medical Biotechnology, School of Advanced Medical Sciences and Technologies, Shiraz University of Medical Sciences, Shiraz, Iran; 10 Laboratory of Basic Sciences, Mohammad Rasul Allah Research Tower, Shiraz University of Medical Sciences, Shiraz, Iran; 11 Department of Clinical Biochemistry, School of Medicine, Kerman University of Medical Sciences, Kerman, Iran; 12 Department of Biochemistry, Fasa University of Medical Sciences, Fasa, Iran; 13 Health Policy Research Center, Institute of Health, Shiraz University of Medical Sciences, Shiraz, Iran

**Keywords:** Brucellosis, Diagnosis, LSPR, Nanobiosensor

## Abstract

Brucellosis is considered as the most common bacterial zoonosis in the world. Although the laboratory findings are the most reliable diagnosis today, the current laboratory methods have many limitations. This research aimed to design and evaluate the performance of a novel technique based on the localized surface plasmon resonance (LSPR) to eliminate or reduce existing shortcomings. For this purpose, smooth lipopolysaccharides were extracted from *Brucella melitensis* and *Brucella abortus* and fixed on the surface of the gold nanoparticles through covalent interactions. After some optimizing processes, dynamic light scattering was used to characterize the probe. The detection of captured anti-Brucella antibody was performed by measuring the redshift on LSPR peak followed by the determination of cutoff value, which indicated a significant difference between controls and true positive patients (*P *value < 0.01). Furthermore, 40 sera from true negative samples and positive patients were used to evaluate the performance of this method by comparing its outcomes with the gold standard (culture), standard tube agglutination test, and anti-brucellosis IgM and IgG levels (ELISA). The sensitivity, specificity, positive predictive value, and negative predictive value showed an appropriate performance of the LSPR-based method (85%, 100%, 100%, and 86%, respectively). The current research results provide a promising fast, convenient, and inexpensive method for detecting the anti-Brucella antibodies in human sera, which can be widely used in medical laboratories to diagnose brucellosis quickly and effectively.

## Introduction

Brucellae are slow-growing Gram-negative coccobacilli belonging to the Brucellaceae family [1]. Brucellae include facultative intracellular bacteria that infect a variety of domestic and wild animals [2]. The discovery of new types of brucellae in recent years has greatly expanded the genus. There are currently twelve species of the genus, four of which include *Brucella. melitensis*, *B. abortus*, *B. suis*, and *B. canis* are the main causes of the disease in humans [3]. *B. melitensis* is considered to be the most virulent species in humans [3]. Even though brucellosis does not cause death in people and is only an exceptional case of person-to-person transmission, the unremitting epidemic of human brucellosis worldwide leads to serious public health concerns and economic damage through the loss of animals productivity. Importantly, the weakening potentiality of brucellosis in humans and the complicated treatment protocol of the disease have made these bacteria candidate agents of biowarfare [4, 5].

Brucellosis in humans is labeled as the ‘disease of mistakes’ [6] because its clinical presentation is nonspecific and overlaps with a wide spectrum of other diseases; hence, laboratory corroboration of the diagnosis is essential for the correct treatment of a patients [7, 8]. Although culture, serological tests, and nucleic acid amplification testing can make the diagnosis of brucellosis, these methods have many limitations. In the culture, as the gold standard, the main limitation that delays the patient's proper diagnosis and treatment is the long incubation time. Advanced automated blood culture systems (e.g., Bactec and BacTAlert systems) can detect acute cases of brucellosis, but they require 5 to 7 days of incubation, and even the incubation and performance of blind subcultures for protracted samples need to be extended [9]. The lack of specificity and false positive results especially in individuals repeatedly exposed to Brucella organisms are the main limitations regarding serological tests [9, 10]. Although they have excellent sensitivity, specificity, safety, and rapid diagnosis of the disease by nucleic acid amplification assays, the clinical importance of these methods and their limited therapeutic implications are not clear due to the long-term perseverance of positive biomolecular test results in patients that have completely recovered [9, 11]. Therefore, it is necessary to design a method that can overcome all test's limitations mentioned above, and at the same time, enhance their advantages.

The improvement of biosensing devices with low detection limits has become an important component of biomarkers detection researches because in the last decade a large number of studies have been dedicated to find appropriate procedures to improve the sensitivity of different detection platforms in biosensing [12, 13]. Biosensors are used to detect and quantify of an analyte by generating a signal from interactions that include biological components. Recently, the increased sensitivity of optical transducers combined with the incomparable specificity of the biomolecular interactions had led to the development of many different optical biosensors [14]. Due to the ease of application, sensitivity to low temperature, and reliable signal generation as evidenced by a redshift in absorption band in response to biological interactions, the tests that are based on the unique optical properties of nanoparticles are cost effective for detecting biological markers [15–17]. These detection methods use the biophysical characteristics of molecules like molecular weight, charge, and refractive index to monitor the activity of a particular molecule. Surface plasmon resonance is a phenomenon caused by the collective oscillation of surface electrons after exposure to incident light that has been used to detect surface-bound biomolecules quickly and easily [18, 19]. SPR consists of two main methods, localized (LSPR) or propagating (PSPR) [20, 21]. Among them, optical biosensors based on localized surface plasmon have developed remarkably because of their significant application potential [12, 22]. This technique comes from the interaction between surface electrons of conductive nanoparticles, which are shorter than the incident light wavelength, and the light wave occurring when in the conduction band incident light interacts with surface electrons [23, 24]. Compared to other similar platforms, The localized surface plasmon resonance based biosensors have several advantages (e.g., surface plasmon resonance), such as having a shorter electromagnetic field decay length, being insensitive to bulk refractive index alterations caused by temperature variations or the components of the surrounding medium, and capability of being excited by freely propagating light [12]. Therefore, in nanobiosensor technology, LSPR-based nanobiosensors are considered to be one of the most powerful tools for detecting biomolecules.

In short, on the one hand, laboratory-based strategies are still the main basis for the diagnosis of brucellosis. On the other hand, current clinical methods face many limitations. Therefore, proposing a new alternative method that can overcome the shortcomings of existing techniques is considered to be one of the main components of treatment protocols. Therefore, this study aimed to introduce a fast, convenient, inexpensive, safe, and sensitive LSPR-based nanobiosensor to detect anti-Brucella antibodies in biological samples for diagnosis of brucellosis. For this purpose, the lipopolysaccharides (LPS) of B. melitensis and B. abortus were coated on gold nanoparticles (GNPs) and the specificity, sensitivity, positive predictive value (PPV), and negative predictive value (NPV) of the technique were evaluated by comparing the results achieved with versus serum culture assay. Furthermore, the current study performed an enzyme-linked immunosorbent assay to measure the anti-Brucellae antibodies (both IgM and IgG) and standard tube agglutination test to assess the ability of currently designed technology to detect brucellosis as opposed to the conventional methods.

## Methods

### Bacterial Culture and LPS Extraction

The smooth strains of *B. melitensis* and *B. abortus* were cultured in *Luria–Bertani* [3] agar. In the next step, bacteria were harvested and LPS were extracted using a modified hot phenol water method [25]. To confirm the quality of extracted LPS, sodium dodecyl sulfate–polyacrylamide gel electrophoresis (SDS-PAGE, Sigma-Aldrich, St. Louis, Missouri, USA) with silver nitrate staining was applied. LPS quantification was performed using 1,9-dimethyl methylene blue (Sigma-Aldrich, St. Louis, Missouri, USA) using *Salmonella typhimurium* as a standard. To assess the protein and nucleic acid contamination, the Bradford method and absorbance at 260 nm were used.

### Synthesis of Spherical Gold Nanoparticles

A chemical reduction of gold salt (HAuCl_4_) was used to synthesize the gold nanoparticles which is a rapid, inexpensive, and green synthesis procedure for Au nanoparticles at room temperature [26]. According to the described method, 15 mg of sodium citrate (Sigma-Aldrich, St. Louis, Missouri, USA) was dissolved in 50 ml of distilled deionized water and was kept in an ice bath and mixed on a magnetic stirrer (150 RPM). At the same time, 600 µl of gold salt solution (17.3 mM) was added. Subsequently, 1.2 ml of sodium borohydride solution (20 mM) was added. The solution was mixed for 2 h under similar conditions and then kept at 4 °C for later application. According to previous studies, in this type of synthesis, sodium citrate simultaneously acts as a reducing agent (driving the reduction of AuIII to Au0), capping agent (electrostatically stabilizing the gold nanoparticles colloidal solution), and pH mediator (modifying the reactivity of Au species involved in the reaction). In this assay, the production of a red-colored solution from a yellow-colored solution of HAuCl4 shows the formation of gold in a zero oxidation state.

Scanning electron microscopy (SEM) was used to study the morphology of gold nanoparticles, and Zetasizer NanoZS90 (Malvern Instruments Ltd, Malvern, Worcestershire, UK) was used to assess the size distribution. Dynamic light scattering (DLS), at a scattering angle of 90º, was used as the basic principle for measuring particle size. Zetasizer Nano used a laser with a 633-nm wavelength. With this technique, the spread of particles caused by Brownian motion is measured and converts to size distribution by the Stokes–Einstein relationship [27]. Zeta potential was also applied to determine the electrical surface charge of nanoparticles. The absorption spectra of nanoparticles were recorded by a Cary 500 UVeviseNIR (ultra-violete–visible near infrared) spectrophotometer (Varian, Australia).

### Construction of Nanoprobe (Biosensor)

#### Carboxylation of Gold Nanoparticles

Gold nanoparticles were coated with thioglycolic acid linkers for preparing the surface of GNPs for loading LPS. In short, 1 ml of TGA solution (1 mM) was mixed with 1 ml of gold nanoparticle solution and kept at room temperature for 24 h. For isolation of coated gold nanoparticles, the solution was centrifuged at 12,000 g for 15 min. After that, two washing steps by double-distilled deionized water were used to remove excess TGA. Finally, spectrophotometry was used to assess the coating of thioglycolic acid on the surface of the GNPs.

#### Optimizing Coating of Thioglycolic Acid on Gold Nanoparticles

The coating of TGA was done at different times, including 12, 18, 24, 36, and 48 h to optimize the coating ratio of TGA on gold nanoparticles. Subsequently, optical density was measured and graphed to obtain the best incubation time.

#### Covalent Attachment of LPS to TGA-Modified Gold Nanoparticles

**Fig. 1:**
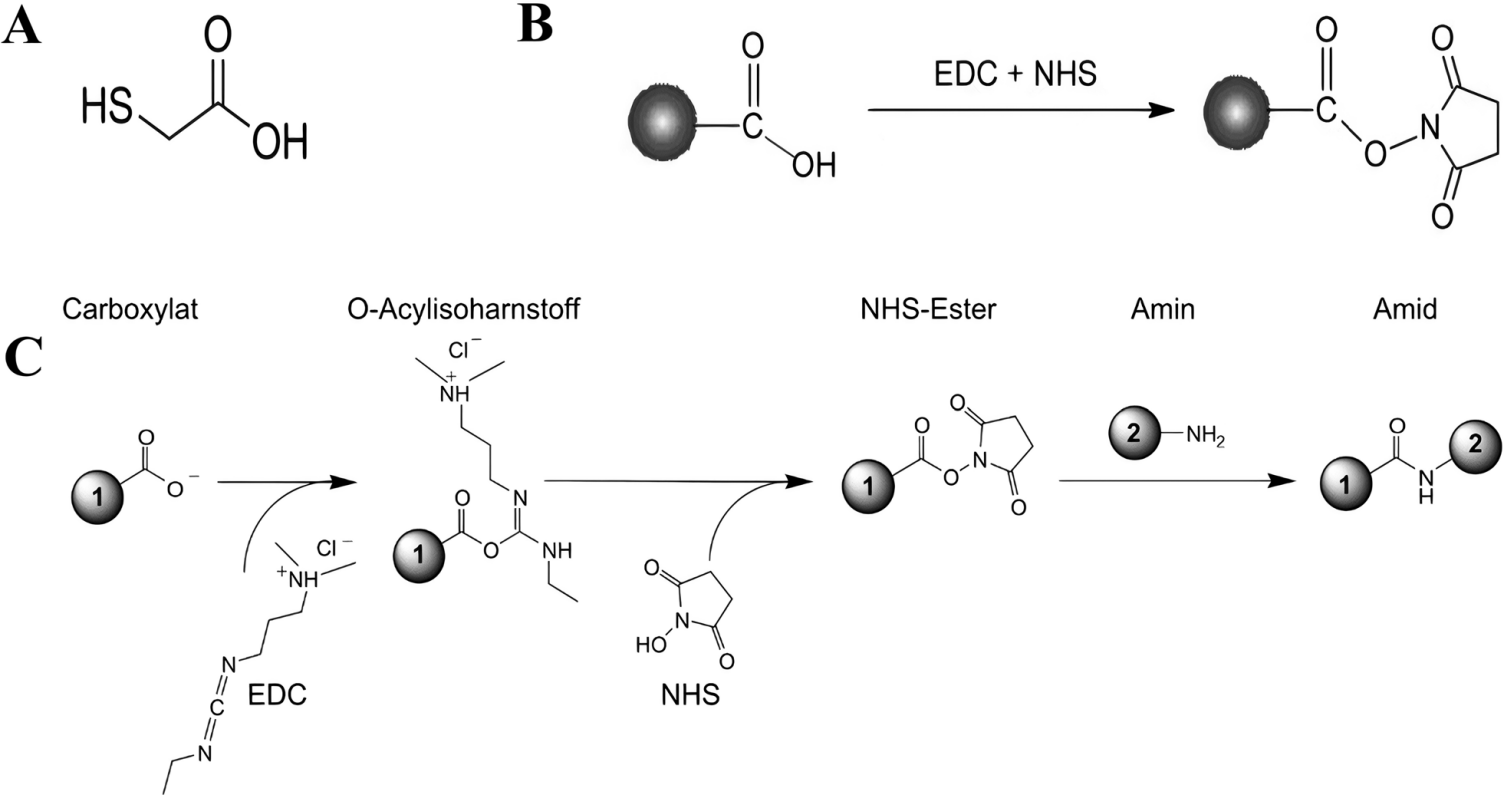
Chemical interactions of LPS attachment to the gold nanoparticles. **A** TGA molecule, **B** EDC + NHS interaction, and **C** Covalent attachment of LPS to TGA-modified gold nanoparticles. Number 1: nanoparticles; number 2: LPS

To stimulate the covalent attachment between the carboxyl group of TGA and the amine group of LPS, the carboxyl groups were activated by EDC (the most popular zero-length crosslinker for biochemical conjugations) and N-hydroxysuccinimide (NHS) molecules [28]. Sedimented gold nanoparticles were suspended in 0.1 mM EDC/NHS solution and incubated for 30 min at room temperature. In the next step, 2 ml of phosphate buffer saline (PBS) containing 0.05% Tween-20 (PBST) (pH 7.4) was added. After a vigorous vortex, nanoparticles were centrifuged at 12,000 RPM for 15 min. After centrifugation, the LPS solution was added following the removal of the supernatant. Subsequently, the mixture was incubated in an ultrasonic bath for 10 min followed by 3 h incubation at room temperature. After that, 2 ml of PBST was added and vortexed vigorously followed by centrifugation for 15 min at 12,000 RPM, and the supernatant was removed. Finally, nanoprobes were then resuspended in 500 µl of PBS and LSPR spectra were measured by spectrophotometer. After the confirmation of LPS attachment to the gold nanoparticles, the solution was kept at 4 °C for further studies. Figure 1 shows the chemical interaction equations the TGA-modified gold nanoparticles synthesis and LPS attachment.

#### Optimizing the LPS Concentration

To maximize the binding of LPS to activated TGA carboxyl groups, optimization of the LPS-to-Au nanoparticle ratio was performed by evaluating the peak shift in LSPR spectra as a function of LPS concentrations (100, 150, 200, 300, and 560 µg/ml) in a fixed amount of the Au nanoparticles.

### Detection of Anti-LPS Antibodies by Nanoprobe

100 µl of the diluted samples (1:50 in PBS) were mixed with 200 µl of the biosensor and mixed by pipetting. In the next step, the biosensors were centrifuged at 12,000 g for 15 min after 30 min of incubation at room temperature. Finally, the biosensors were suspended in 200 µl of PBS, and the absorbance was measured as described previously. To validate the nanobiosensor function, positive and negative controls were also provided from a commercially available ELISA kit (Pishtazteb Zaman, Iran).

### Ethics Statement

The use of human sera was approved by the Ethics Committees of Fasa University of Medical Sciences Fasa, Iran (IR.FUMS.1396.324). Identities of sera donors were coded and not revealed to anyone involved in this study. Furthermore, all procedures were performed under ethical guidelines following national regulations. Moreover, all samples were from subjects who had signed the written informed consent.

### Evaluating the Function of the Designed Method

Forty samples containing 20 cases (true positives) and 20 controls (true negatives) were provided to evaluate the functionality of the designed method. For this reason, the performance of the designed method in the present study was compared with the culture results. Furthermore, available commercial kits (Pishtaz Teb, Tehran, Iran) were used to assess the serum levels of IgM and IgG antibodies. The sample preparation and antibodies evaluation were performed according to the manufacturer's protocol (Specificity: 99.85%, Sensitivity: 99.4%). Additionally, the standard tube agglutination test was performed on all of the sera samples to compare with and validate the performance of the LSPR-based method. To evaluate the performance of the designed method, the obtained results from the LSPR-based method were compared to the mentioned conventional tests with calculating sensitivity, specificity, positive predictive value, and negative predictive value based on the common formula as follows:

$$\begin{aligned} {\text{Sensitivity}} & = \frac{{{\text{Number}}\,{\text{of}}\,{\text{True}}\,{\text{Positives}}}}{{{\text{Number}}\,{\text{of}}\,{\text{True}}\,{\text{Positives}}\, + \,{\text{Number}}\,{\text{of}}\,{\text{False}}\,{\text{Negatives}}}} \\ {\text{Specificity}} & = \frac{{{\text{Number}}\,{\text{of}}\,{\text{True}}\,{\text{Negatives}}}}{{{\text{Number}}\,{\text{of}}\,{\text{True}}\,{\text{Negatives}}\, + {\text{Number}}\,{\text{of}}\,{\text{False}}\,{\text{Positives}}}} \\ {\text{PPV}} & = \frac{{{\text{Number}}\,{\text{of}}\,{\text{True}}\,{\text{Positives}}}}{{{\text{Number}}\,{\text{of}}\,{\text{True}}\,{\text{Positives}}\, + \,{\text{Number}}\,{\text{of}}\,{\text{False}}\,{\text{Positives}}}} \\ {\text{NPV}} & = \frac{{{\text{Number}}\,{\text{of}}\,{\text{True}}\,{\text{Negatives}}}}{{{\text{Number}}\,{\text{of}}\,{\text{True}}\,{\text{Negatives}}\, + \,{\text{Number}}\,{\text{of}}\,{\text{False}}\,{\text{Negatives}}}} \\ \end{aligned}$$

### Statistical Analysis

This study was analyzed as a classic diagnostic performance experiment by evaluating the agreement of a proposed test, the LSPR-nanosensor, and a reference standard test, the culture, and some conventional tests including ELISA and SAT to determine their ability for identifying a target condition. Cutoff values were calculated by the average of controls (true negative) sera plus two-time standard deviation values (2SD). The Kolmogorov–Smirnov test was performed to determine the normality and evaluate the data distribution. Based on the normality outcomes, which revealed the parametric distribution, the ANOVA test was used to compare groups. All statistical analyses were performed using GraphPad Prism for windows version 8 and SPSS for windows version 9.

## Results

### Lipopolysaccharides Extraction Analysis

SDS-PAGE showed that the yield of LPS extraction was approximately 1 percent of the used bacteria wet weight. Moreover, the concentration of nucleic acid was ≤ 0.2% of LPS concentration. Importantly, the Bradford method demonstrated the absence of any protein contaminations.

### Characterization of Gold Nanoparticles

**Fig. 2:**
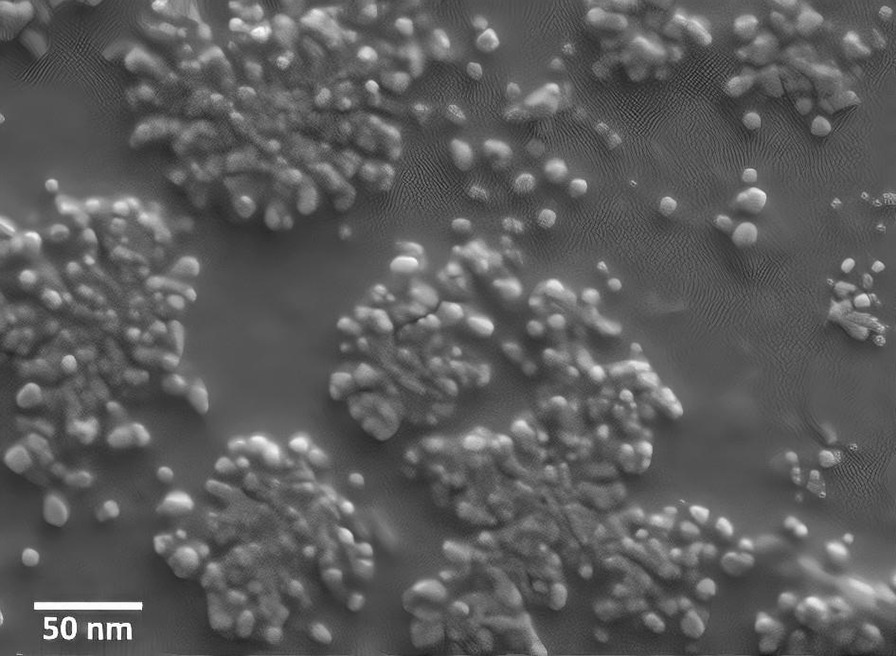
Structure of gold nanoparticles under SEM. The equal distribution of gold nanoparticles is well-reflected in the figure

The size distribution of the nanoparticles determines the quality of a biosensor. According to the previous studies, nanoparticles must possess a specific size. In this regard, the appropriately small size of the nanoparticles results in suitable colloidal stability, high surface-to-volume ratios, and rapid movement for high binding rates results in high affinity, high sensitivity, and high selectivity in interaction with biological targets. Furthermore, the nanoparticles must be as large as possible to allow the presents of various ligands on the surface of the particle and obtain multivalent interactions, too [29–31]. As Gu et al. reported, the comparability of the size of nanoparticles with the size of biological targets is particularly important in the interaction of proteins [32]. According to the size of anti-Brucella antibodies, which is 2–5 nm [33], this study prepared gold nanoparticles with an average size of 10 nm and performed the determination of the nanoparticles size distribution by a Zetasizer NanoZS90 instrument, which uses a laser with a 633-nm wavelength. This technique benefits from the Stokes–Einstein equation which can convert particles diffusion, caused by Brownian motion into a size distribution. According to Fig. 2, the GNPs were uniformly distributed at a 10-nm scale. Zeta potential values revealed details about the surface charge and stability of the GNPs. The particles were negatively charged, and their zeta potential was approximately − 28 mV. Furthermore, Table 1 shows the maximum absorbance wavelength of GNPs. Visual and ultraviolet wavelengths were measured by spectrophotometer, and maximum absorbance was 530 nm. We used the Turkevich method to produce gold nanoparticles. The advantages of this method is the production of gold nanoparticles including easy and cheap production process, ability to control the size of nanoparticles, and proper colloidal stability [34].

**Table 1:** Properties of GNPs

Mean size	Maximum absorbance	Maximum peak intensity	Zeta potential
10 nm	530 nm	0.880	− 28

As the table illustrates, the size of gold nanoparticles was distributed equally. Furthermore, the table provides information about the absorbance and intensity of GNPs

### Characterization of the Nano-bio-Probe

**Fig. 3:**
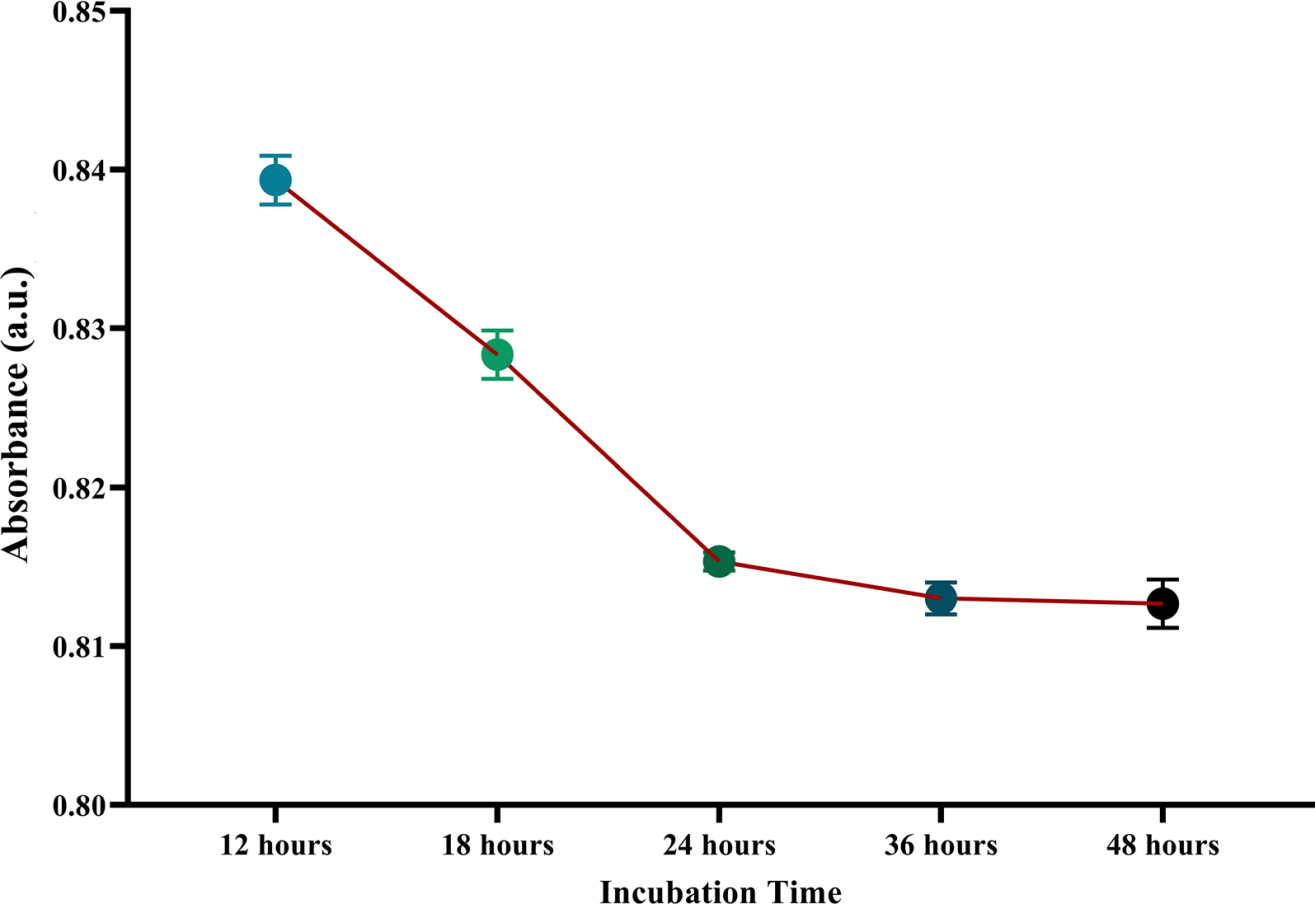
Optimizing coating of thioglycolic acid on gold nanoparticles. As the figure reveals, there is no significant alteration in absorbance after 24 h of incubation despite the elongation of adjacent time. Therefore, 24 h was selected as the optimized time for coating of thioglycolic acid on GNPs. The *Y*-axis shows the absorbance intensity at 530 nm (max absorbance of nanoparticles). The experiments were done in triplicates. Data are presented as mean ± SEM. a.u.: Absorbance unit

As mentioned above, this study used TGA molecules to connect the gold nanoparticles with LPS resulting in a slight 4.55% decrease in the LSPR peak at 530 nm. Besides, the present study performed several incubation times to determine the maximum TGA adherence to the gold nanoparticles. According to the results shown in Fig. 3, 24 h of TGA incubation with GNPs resulted in the maximum TGA coating on the surface of the gold nanoparticles.

**Fig. 4:**
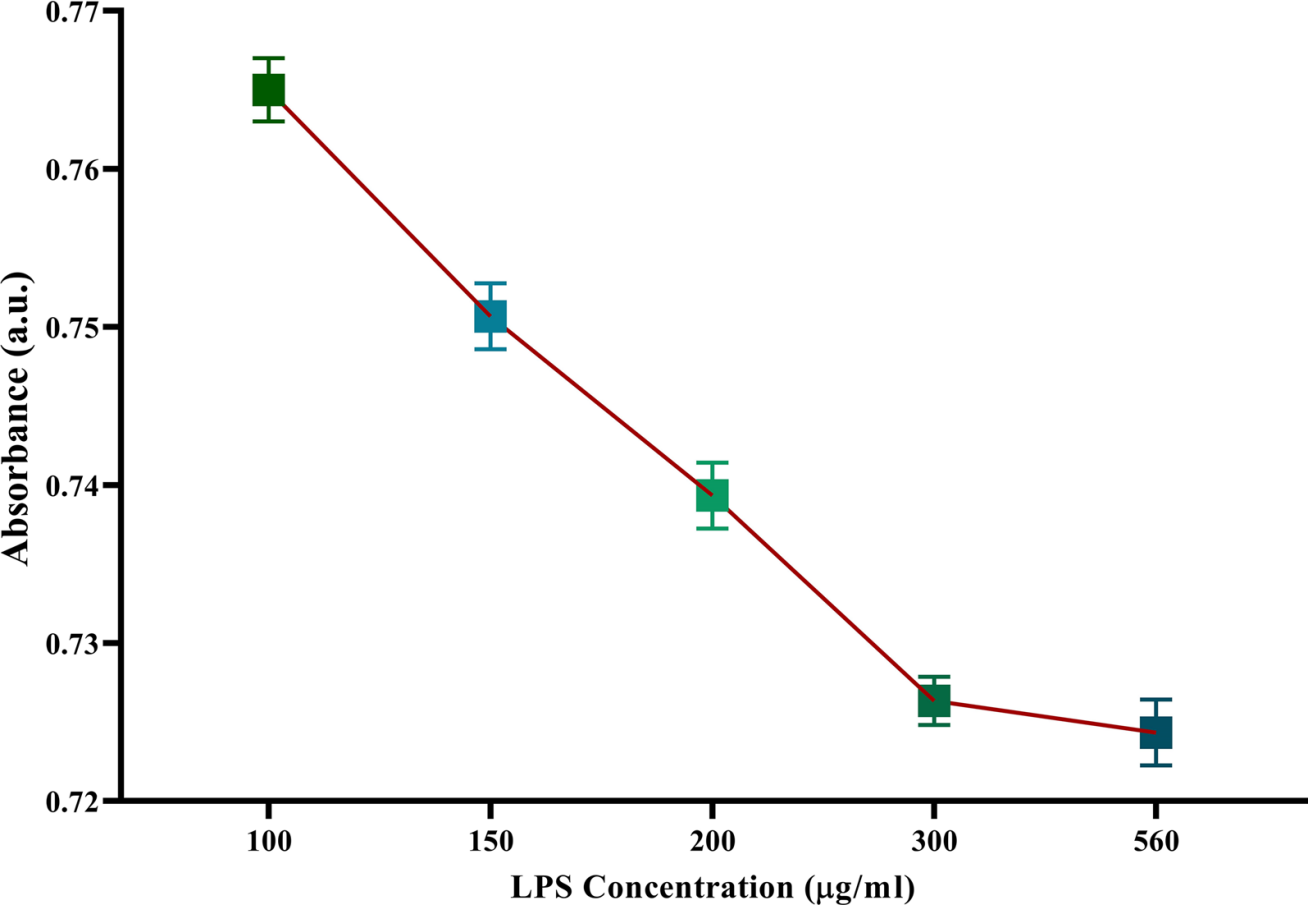
Optimizing the LPS concentration. As the concentration of LPS increased, the absorbance decreased significantly. However, upon the increment in LPS concentrations of more than 300 mg/ml, there was no significant reduction in the LSPR absorbance. Hence, the mentioned concentration was selected as the optimized concentration. The *Y*-axis shows the absorbance intensity at 530 nm (max absorbance of nanoparticles). The experiments were done in triplicates. Data are presented as mean ± SEM. a.u.: Absorbance unit

Regarding the determination of the optimized concentration of LPS, the present study investigated concentrations including 100, 150, 200, 300, and 560 ug/ml. As shown in Fig. 4, as the concentration had increased, the absorbance decreases. According to the results, the present study selected a 300 ug/ml concentration of LPS to coat the surface of TGA-modified GNPs, because there was not any notable decrease in absorption peak intensity by increasing the LPS concentration.

### Assessment of the Nano-bio-Probe Functions

**Fig. 5:**
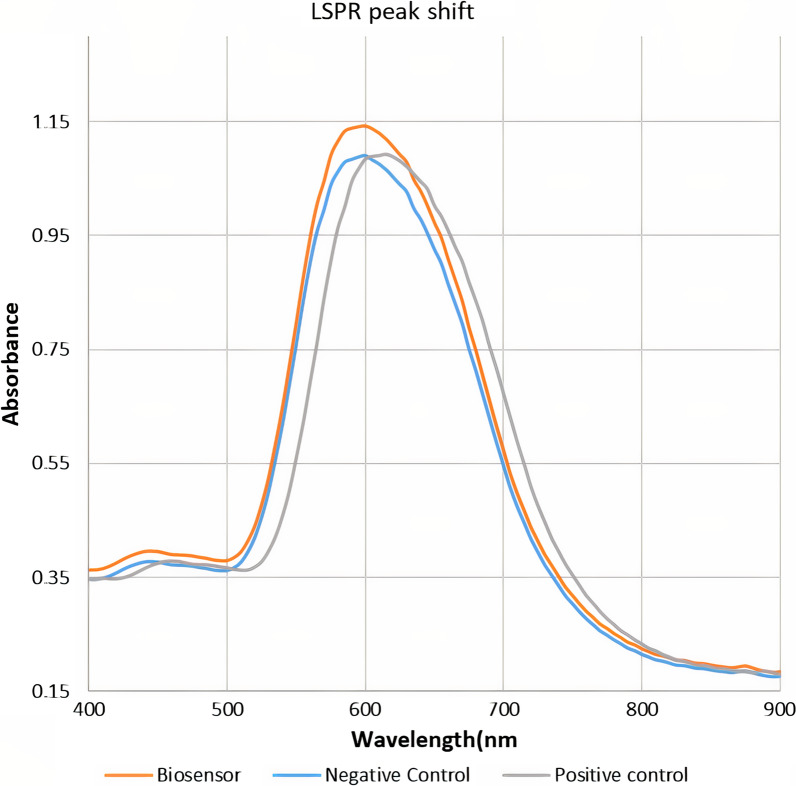
Peak of LSPR nanoprobe in the presence of positive and negative controls. Controls were provided from commercially available ELISA kits

As the LSPR technique is a surface-sensitive optical method, it can detect the interaction between anti-LPS antibodies and antigens in the surface of the gold nanoparticles through the image of alterations in the LSPR absorption peak which is caused by electrostatic interaction between antibody and antigen. To confirm the correct functioning of the biosensor, this study prepared positive control comprised of anti-Brucella LPS antibodies with a titer of 1:80 and negative control, and recorded the LSPR spectrum of biosensor after incubation with the controls. As shown in Fig. 5, incubation with negative control did not affect the absorbance of LSPR. However, incubation with positive control caused a shift in the LSPR maximum absorbance which was formerly known as redshift. Therefore, the binding of anti-Brucella antibodies to the LPS antigens limits the incidence of hitting light on the surface of nanoprobes.

### Determining Cutoff Value

**Fig. 6:**
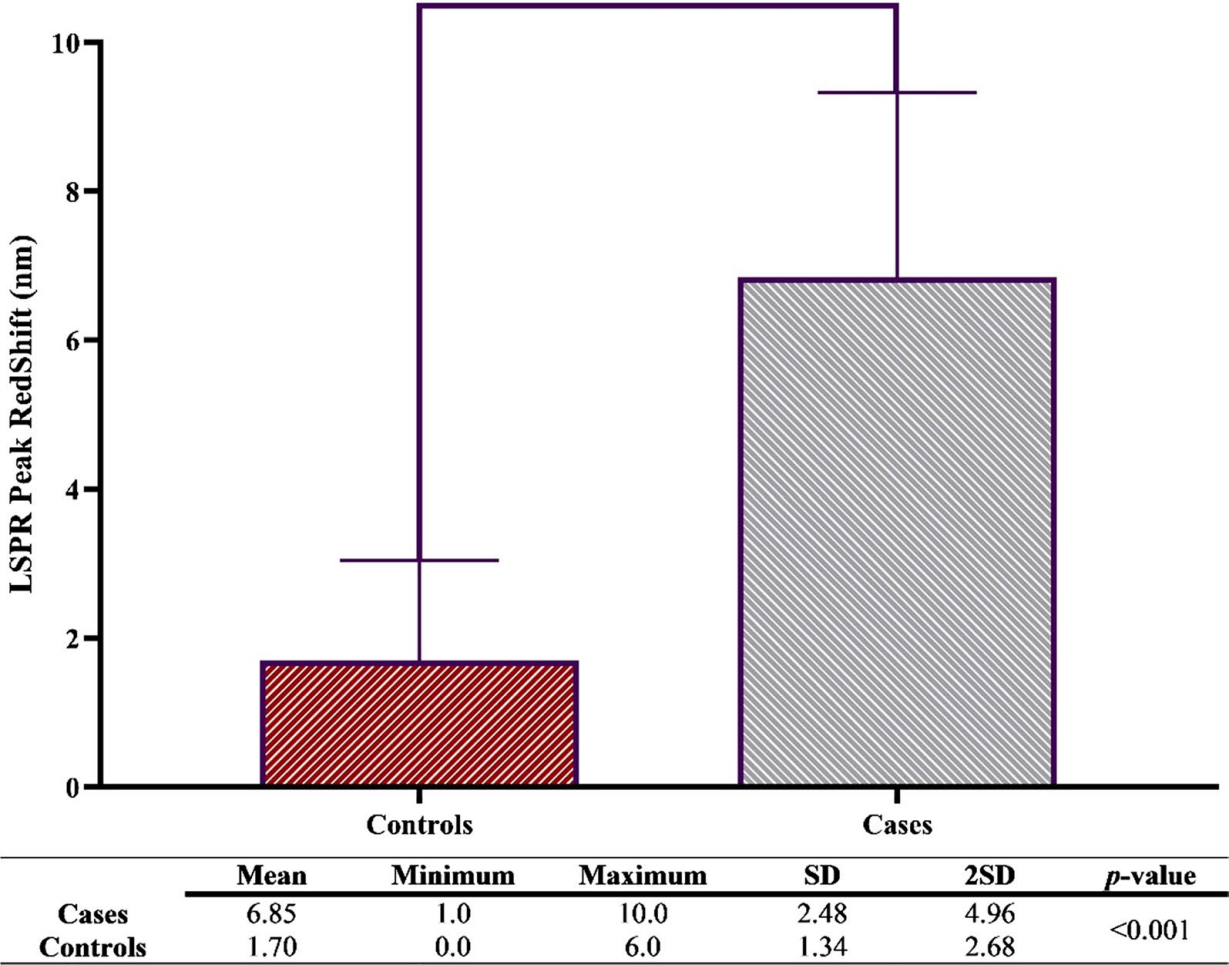
LSPR peak redshift in the sera of truly patients and control subjects. Upon the interaction between antibodies, which presented in the sera of infected individuals, a redshift took place that was significantly different when compared to controls. Furthermore, the figure demonstrates the SD and 2SD values that are precious for the definition of the cutoff point. The experiments were done in triplicates for each sample. *P *value < 0.05 was considered significant

For this purpose, this study obtained 40 sera from true positive and negative subjects based on the culture and clinical observations. As shown in Fig. 6, the redshift average in true negative samples (controls) was 1.70 nm which was significantly different from true positive patients (*P* value < 0.001). Furthermore, the results indicated that the redshift of 4.38 nm should be used as the cutoff value. Interestingly, only 5% of controls had a redshift above cutoff value, and the redshift in all patients was above the cutoff value indicating that the performance of the method is acceptable and the determination of cutoff value is reliable.

### Validation of Biosensor Function

In the previous steps, this study introduced a rapid and inexpensive method to identify anti-Brucella antibodies in the samples. However, verifying the functionality of a new method is necessary. For this aim, this study conducted a series of sub-studies including standard tube agglutination test, anti-IgG, and anti-IgM levels in the sera of negative and positive samples based on the culture results. As shown in Table 2, the sensitivity of the current method was less than that of SAT alone. Importantly, the specificity of the LSPR-based method showed the most acceptable result which was 100%. Similar to specificity, the positive predictive value of the current method was higher than other conventional tests (100%). Ultimately, the NPV of SAT, IgG, IgM and LSPR-based methods were 90%, 90%, 81%, and 86%, respectively.

**Table 2:** Validation of the LSPR-based method by comparing its performance with conventional tests

Methods	Positive	Negative	Sen (%)	Spc (%)	NPV (%)	PPV (%)
A. Gold Standard	20	20	–	–	–	–
B. SAT						
Positive	18	1	90	95	90	94
Negative	2	19				
C. IgG						
Positive	17	1	85	95	90	89
Negative	3	19				
D. IgM						
Positive	16	2	80	90	91	88
Negative	4	18				
E. LSPR						
Positive	17	0	85	100	86	100
Negative	3	20				

The obtained results emphasize the appropriate and comparable performance of the designed method

Sen, Sensitivity; Spc, Specificity; NPV, Negative Predictive Value; PPV, Positive Predictive Value

## Discussion

Brucellosis is probably considered to be the first human infection after the domestication of cattle, sheep, and goats [35]. Brucellosis is the world’s most prevalent bacterial zoonosis with 500,000 new human cases of the disease diagnosed globally each year [9, 36]. The symptoms of human brucellosis are not pathognomonic because the infection may affect any organ of the body [37]. Although the laboratory findings are still a reliable factor in disease detection, the current strategies including culture, serology, and nucleic acid amplification tests have shown serious flaws [9, 38–40]. Therefore, this study attempts to introduce a new diagnostic method based on LSPR to remove the limitations.

In recent years, the localized surface plasmon resonance has been used as the basis for the development of biosensors. The advantages of current research on gold nanoparticles and advances in nanotechnology have been considered the main factors to improve diagnostic performance. The unique color and optical properties of GNPs, due to their plasmonic and adjustable effects, are two outstanding and beneficial characteristics of these particles compared to other particles [12, 41].

In this study, a redshift in the LSPR peak was used as an indicator of interaction between LPS antigens and anti-Brucella antibodies. In the other words, compared to the control samples, the presence of antibodies in the serum of the case samples caused a significant redshift of the LSPR absorption peak which can be assumed to be an indicator of brucellosis. Although there is a significant difference in redshift between control and case samples, this difference cannot quantify the concentration of antibodies in the serum, which can be considered as one of the serious limitations of this method. However, we must remember that some common methods, such as ELISA-related strategies, are usually considered as a cut-off point [42, 43].

Importantly, this study validated the performance of the LSPR-based technique by comparing the obtained results with other current methods by evaluating various parameters. The first analyzed parameter was sensitivity which determines the probability of a positive brucellosis test in a patient [44]. As Yagupsky et al. had discussed [9], the sensitivity of blood culture, which is considered as the current gold standard, is reduced especially in chronic disease and focal infections. In addition, this method also faced other serious limitations, such as laboratory safety problems, the slow-growth of bacteria, and a positive result is indisputable evidence of the disease [45, 46]. This LSPR-based technique represented a reliable and competitive result compared to the conventional methods in sensitivity such as culture, SAT, and ELISA-dependent kits. At the same time, specificity, that is the probability of a negative test if the patient is not infected, was evaluated [45, 47]. As results showed, compared to the other diagnostic strategy, the designed LSPR-based technique had the most significant outcome in terms of specificity. It is an important characteristic of this method because the previous studies had questioned the validity and specificity of serodiagnostic methods of human brucellosis since asymptomatic and self-limiting episodes of this infection occur in zoonosis endemicity areas [48–50]. However, like antibody-related methods, the current method resists the possible long-term persistence of anti-Brucella antibodies within the serum, even after complete recovery from brucellosis.

Similar to previous studies, positive predictive value was defined as the ratio of the true positive results to the sum of all positive results [51]. Similarly, the NPV was described as the ratio of the true negative results to the sum of all negative results [51]. Compared to conventional diagnostic strategies, the current designed method represented the most wonderful PPV outcomes, demonstrating its ability to distinguish between infected and non-infected individuals. In addition, the evaluation of the NPV results revealed the promising potential of the LSPR-based method to exclude non-patients from real patients.

## Conclusion

This study introduced a rapid, simple, low cost, reliable, and economical method for diagnosing brucellosis. Although there are some minor limitations, including the qualitative outcomes and the possibility of being positive in recovered individuals, the advantages discussed show that it has good capabilities compared to the current laboratory-based diagnostic procedures.

## Data Availability

The datasets used and/or analyzed during the current study are available from the corresponding author on reasonable request.

## Abbreviations

LPS: Lipopolysaccharides
GNPs: Gold nanoparticles
DLS: Dynamic light scattering
LSPR: Localized surface plasmon resonance
SAT: Standard tube agglutination test
ELISA: Enzyme-linked immunosorbent assay
PPV: Positive predictive value
NPV: Negative predictive value
PSPR: Propagating surface plasmon resonance
SDS-PAGE: Sodium dodecyl sulfate–polyacrylamide gel electrophoresis
SEM: Scanning electron microscopy
TGA: Thioglycolic acid
NHS: N-hydroxysuccinimide
EDC: *N*-(3-dimethylaminopropyl)-*N*′-ethylcarbodiimide hydrochloride
PBS: Phosphate buffer saline
